# Comparison of thoracolumbar versus non-thoracolumbar osteoporotic vertebral compression fractures in risk factors, vertebral compression degree and pre-hospital back pain

**DOI:** 10.1186/s13018-023-04140-6

**Published:** 2023-08-30

**Authors:** Feng Wang, Rui Sun, Shao-Dong Zhang, Xiao-Tao Wu

**Affiliations:** 1 Department of Spine Surgery, Zhongda Hospital, School of Medicine, Southeast University, 87# Dingjiaqiao Road, Nanjing, 210009 China; 2 Surgery Research Center, School of Medicine, Southeast University, 87# Dingjiaqiao Road, Nanjing, 210009 China

**Keywords:** Osteoporotic vertebral compression fracture, Spine trauma, Thoracolumbar spine, Back pain, Bone fragility

## Abstract

**Background:**

Thoracolumbar spine is at high risk of osteoporotic vertebral compression fractures (OVCF). This study aimed to identify the differences in risk factors, vertebral compression degree and back pain characteristics of thoracolumbar OVCF (TL-OVCF) and non-thoracolumbar OVCF (nTL-OVCF).

**Methods:**

OVCF patients hospitalized in a spine center between June 2016 and October 2020 were retrospectively studied. Demographics, comorbidity, spine trauma, bone mineral density, duration of pre-hospital back pain, extent of vertebral marrow edema, and degree of vertebral compression of patients with nTL-OVCF were summarized and compared to those with TL-OVCF.

**Results:**

A total of 944 patients with acute single-segment OVCF were included. There were 708 (75.0%) TL-OVCF located in T11-L2 and 236 (25.0%) nTL-OVCF in lower lumbar (L3-L5) and middle thoracic (T5-T10) spine. The female-male ratio was 4.1 in nTL-OVCF and differed not significantly from TL-OVCF. The middle thoracic OVCF were older and had higher comorbidity of coronary heart disease (21.3%) and cerebral infarction (36.3%) than TL-OVCF (12.1% and 20.6%). In nTL-OVCF the ratio of apparent spine trauma (44.9%) and pre-hospital back pain ≤ 1 week (47.5%) was lower than in TL-OVCF (66.9% and 62.6%). The T-score value of lumbar spine was − 2.99 ± 1.11, − 3.24 ± 1.14, − 3.05 ± 1.40 in < 70, 70–80, > 80 years old TL-OVCF and differed not significantly from nTL-OVCF. The lower lumbar OVCF had more cranial type of vertebral marrow edema (21.8%) and fewer concurrent lumbodorsal fasciitis (30.8%) than TL-OVCF (16.8% and 43.4%). In TL-OVCF the anterior–posterior vertebral height ratio was lower with back pain for > 4 weeks than for ≤ 1, 1–2, and 2–4 weeks. In nTL-OVCF the degree of vertebral compression differed not significantly with pre-hospital back pain for ≤ 1, 1–2, 2–4, and > 4 weeks.

**Conclusions:**

Thoracolumbar spine has 2-folds higher risk of OVCF than non-thoracolumbar spine. Non-thoracolumbar OVCF are not associated with female gender, apparent spine trauma or poor bone mineral density, but tend to maintain the degree of vertebral compression and cause longer duration of pre-hospital back pain.

## Background

Osteoporotic vertebral compression fractures (OVCF) are common in aged population with bone fragility, causing back pain and promoting kyphosis [1, 2]. The worldwide incidence of OVCF was estimated to be 1.4 million per year [3], which most often involved thoracolumbar spine [4]. Biomechanical modeling has revealed that the junction of thoracic and lumbar spine yields mechanical stress on different patterns of loading [5–9], thus increasing the risk of thoracolumbar OVCF (TL-OVCF). On the other hand, mechanical support from rib cage and paravertebral muscles might help to reduce the risk of non-thoracolumbar OVCF (nTL-OVCF) in middle thoracic and lower lumbar spine [5, 9, 10]. In addition to bone fragility and mechanical stress, evidence accumulates that OVCF are associated with multiple risk factors ranging from aging, female gender, comorbidity of cardiovascular and cerebrovascular diseases, to life styles such as chronic smoking and alcohol consumption [11–14]. However, to date, it is unknown whether TL-OVCF and nTL-OVCF would have different risk factors and clinical manifestation of OVCF. We hypothesize in the non-thoracolumbar spine with comparatively lower fracture risk, OVCF might be associated with severer spine trauma or poorer bone fragility than that in TL-OVCF.

In this study, the demographics, comorbidity profiles, spine trauma, bone mineral density, duration of pre-hospital back pain, extent of vertebral marrow edema, and degree of vertebral compression of patients with nTL-OVCF were summarized and compared to those with TL-OVCF. By identifying the differences in risk factors and back pain characteristics of OVCF within and beyond the thoracolumbar spine, our study may help to provide a segment-specific approach to better understand the pathogenesis and clinical manifestation of OVCF.

## Methods

### Study population

**Fig. 1:**
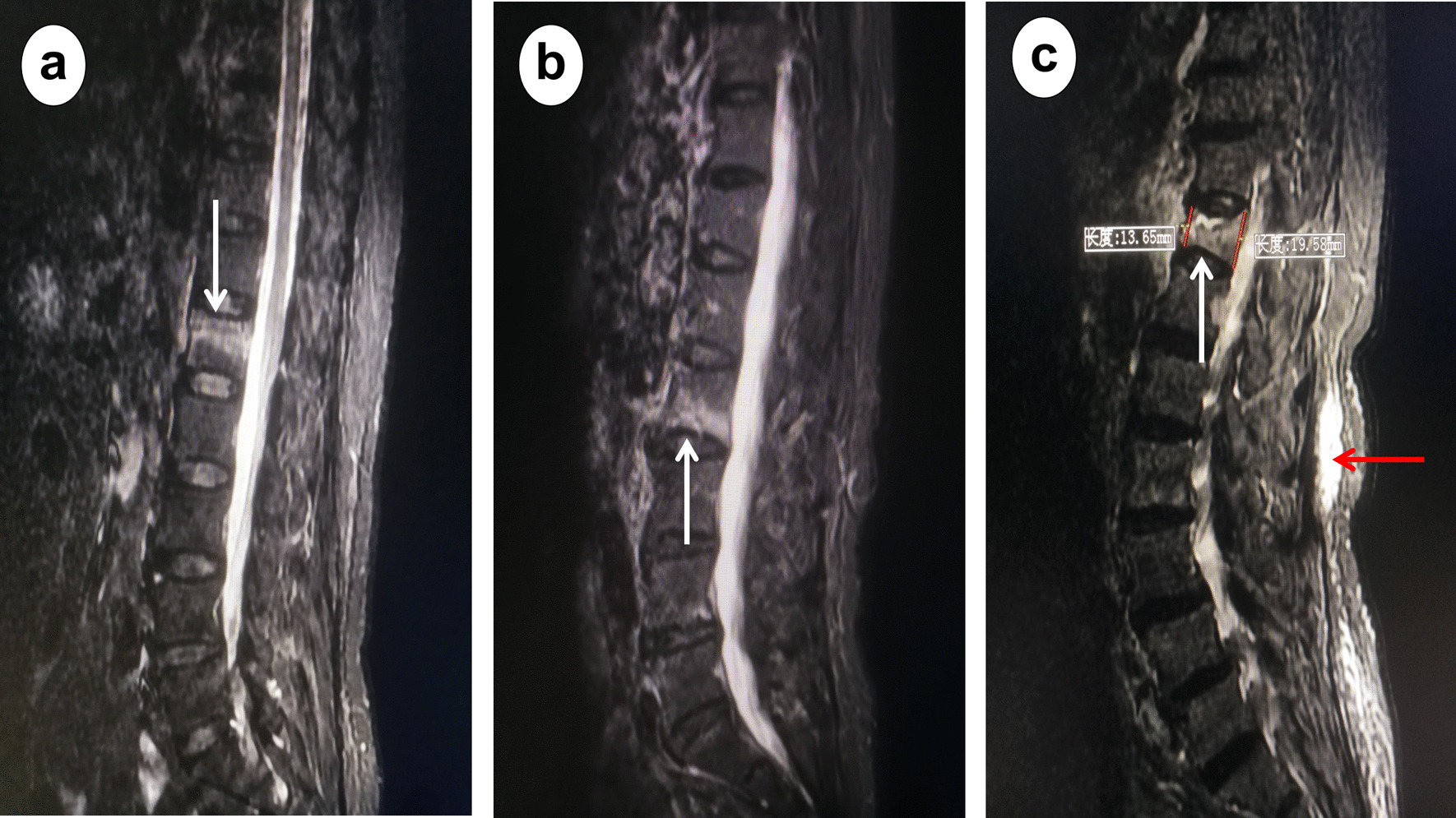
Extent of vertebral bone marrow edema and degree of vertebral compression. The sagittal T2-weighted fat suppression MRI of lumbar spine (**a-c**). A 72 years old female complaining back pain for 1 day after fell on ground showed vertebral bone marrow edema (white arrow) in the cranial half of L1 vertebrae (**a**). A 76 years old female complaining back pain for 10 days after fell on ground showed vertebral bone marrow edema in the caudal half of L2 vertebrae (**b**). A 67 years old female complaining back pain for 11 days after fell on ground showed vertebral bone marrow edema in both cranial and caudal half of T12 vertebrae (**c**). Longitudinal edema signals (red arrow) were detected along and dorsal to thoracolumbar fascia (**c**). The anterior and posterior height (red line) of T12 vertebrae was measured to quantify the degree of vertebral compression (**c**)

The study was approved by Ethic Committee for Clinical Research of Zhongda hospital affiliated to Southeast University (No.2022ZDSYLL016). Medical records and magnetic resonance imaging (MRI) of OVCF patients received vertebroplasty or kyphoplasty in the spine center from June 2016 to October 2020 were retrospectively studied. For accurate defining thoracolumbar and non-thoracolumbar OVCF, only the single-segment OVCF were included in this study. Inclusion criteria: (1) aged ≥ 45 years old; (2) diagnosis of single-segment OVCF based on complains of back pain and MRI of bone marrow edema in single thoracic or lumbar vertebrae (Fig. 1); (3) full medical records depicting the spine trauma, comorbidity, and duration of back pain before hospitalization. Exclusion criteria: (1) diagnosis of vertebral infection, hemangioma, multiple myeloma, metastatic tumors, and other pathological vertebral fractures; (2) previous spine surgery with vertebroplasty or internal fixation; (3) incomplete medical records or MRI of thoracic and lumbar spine.

### Grouping and data collection

The study population was grouped according to the anatomical location and incidence of OVCF: group of TL-OVCF that yielded high incidence of OVCF involving T11-L2 vertebra, and group of nTL-OVCF that yielded comparatively lower incidence of OVCF in middle thoracic (T5-T10) and lower lumbar (L3-L5) spine. In case of lumbosacral transitional vertebra, two radiologists identified and reconfirmed the location of OVCF.

In each group the demographics (age, gender), comorbidity profiles (hypertension, diabetes mellitus, coronary heart disease, cerebral infarction, and chronic obstructive pulmonary disease), spine trauma, and duration of pre-hospital back pain were summarized from medical records. The age of patient was sub-grouped into < 60, 60–70, 70–80, and ≥ 80 years old. The type of spine trauma was divided into apparent trauma: fall on ground or crush injury to spine; uncertain trauma: heavy lift injury, lumbar sprain, strenuous cough; and no evident trauma. The duration of pre-hospital back pain was sub-grouped into ≤ 1 week, 1–2 weeks, 2–4 weeks, 1–3 months, and > 3 months. Bone mineral density (BMD) was quantified by the T-score values calculated from the dual-energy X-ray absorptiometry (DXA) of lumbar spine and hip joint.

MRI of thoracic and lumbar spine was reviewed to quantify the degree of vertebral compression and extent of bone marrow edema. The anterior and posterior height of compressed vertebrae was measured on the sagittal MRI taken at mid-body and medial wall of two pedicles (Fig. 1). The degree of vertebral compression was quantified by averaging the ratio of anterior and posterior vertebral height. The extent of vertebral marrow edema was evaluated on the sagittal T2-weighted fat suppression MRI and defined as diffused type: edema detected both in the cranial and caudal half of vertebrae; cranial type: edema restrained in the cranial half; caudal type: edema restrained in the caudal half (Fig. 1). In case that longitudinal edema signals were detected along and dorsal to the thoracolumbar fascia, the patient was diagnosed of OVCF with concurrent lumbodorsal fasciitis (Fig. 1).

### Statistical analysis

Prism software (ver.9.1.2; Graphpad, San Diego, CA, USA) was used to perform statistical analysis. Descriptive statistics with Pearson χ2 were performed to compare the frequencies and percentages of categorical variables. Continuous quantitative data were presented as means ± standard deviations. Differences between two groups were analyzed by unpaired *t* test. Differences among multiple groups were analyzed by one-way ANOVA followed by Tukey’s multiple comparisons test. Statistical significance was defined as P value < 0.05.

## Results

### Thoracolumbar spine had 2-folds higher risk of OVCF than non-thoracolumbar spine

**Fig. 2:**
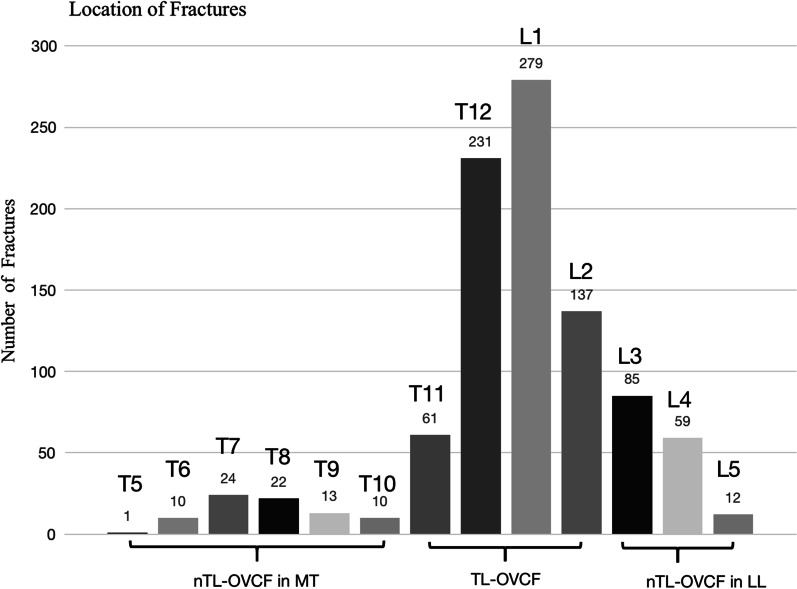
Anatomical distribution of single-segment OVCF. 944 single-segment OVCF showed asymmetrical bimodal distribution pattern peaked at L1 in thoracolumbar spine and T7 in middle thoracic spine. TL-OVCF: thoracolumbar OVCF; nTL-OVCF: non-thoracolumbar OVCF; MT: middle thoracic; LL: lower lumbar

A total of 1490 vertebroplasty or kyphoplasty surgeries were performed in the spine center of Zhongda hospital from June 2016 to October 2020, of which 944 single-segment OVCF were included in this study. The 944 OVCF were unevenly distributed in T5-L5 vertebra, with L1 having the highest incidence (29.5%) of OVCF. The incidence of OVCF decreased gradually from L1 to L5 and from T12 to T10, then increased upwards and peaked at T7, showing an asymmetrical bimodal distribution pattern (Fig. 2). There were 708 (75.0%) TL-OVCF located in T11-L2, 156 (16.5%) lower lumbar OVCF in L3-L5, and 80 (8.5%) middle thoracic OVCF in T5-T10. Thoracolumbar spine had 2-folds higher risk of OVCF than non-thoracolumbar spine.

### nTL-OVCF in middle thoracic spine were older and increased with aging in females

The 944 OVCF were aged 45–97 years old and included 769 females and 175 males. The female-male ratio was 4.1 in nTL-OVCF and differed not significantly from that of 4.5 in TL-OVCF (Table 1). Females were 71.52 ± 9.37 yeas old and on average 3.1 years older than males (74.63 ± 10.29 yeas old; t = 3.88, P < 0.001). nTL-OVCF were aged 72.25 ± 10.10 years old and differed not significantly from TL-OVCF (72.05 ± 9.46 yeas old; t = 0.285, P = 0.776). nTL-OVCF in middle thoracic spine were aged 74.95 ± 9.78 years old and significantly older than lower lumbar OVCF (70.87 ± 10.00 years old; q = 4.38, P = 0.006) and TL-OVCF (q = 3.63, P = 0.028). The ratio of females aged < 60, 60–70, 70–80, and ≥ 80 years old showed steady increase in middle thoracic OVCF but not in lower lumbar OVCF and TL-OVCF (Table 1).

**Table 1:** Clinical characteristics of thoracolumbar and non-thoracolumbar OVCF

	TL-OVCF	nTL-OVCF		*P*-value
		MT	LL	
	(*N* = 708)	(*N* = 80)	(*N* = 156)	
	*N* (%)	*N* (%)	*N* (%)	
Gender				0.776
Female	579 (81.78)	66 (82.50)	124 (79.49)	
Male	129 (18.22)	14 (17.50)	32 (20.51)	
Age for patients (in years)				0.026
< 60	67 (9.46)	6 (7.50)	19 (12.18)	
60–70	225 (31.78)	16 (20.00)	57 (36.54)	
70–80	233 (32.91)	25 (31.25)	45 (28.85)	
≥ 80	183 (25.85)	33 (41.25)	35 (22.44)	
Age for female (in years)				0.026
< 60	60 (10.36)	5 (7.58)	14 (11.29)	
60–70	195 (33.68)	13 (19.70)	48 (38.71)	
70–80	191 (32.99)	22 (33.33)	40 (32.26)	
≥ 80	133 (22.97)	26 (39.39)	22 (17.74)	
Age for male (in years)				0.309
< 60	7 (5.43)	1 (7.14)	5 (15.63)	
60–70	30 (23.26)	3 (21.43)	9 (28.13)	
70–80	42 (32.56)	3 (21.43)	5 (15.63)	
≥ 80	50 (38.76)	7 (50.00)	13 (40.63)	
Type of spine trauma				< 0.001
Apparent trauma	474 (66.95)	26 (32.50)	80 (51.28)	
Uncertain trauma	77 (10.88)	40 (50.00)	55 (35.26)	
No evident trauma	157 (22.18)	14 (17.50)	21(13.46)	
Duration of back pain				< 0.001
≤ 1 week	443 (62.57)	35 (43.75)	77 (49.36)	
1–2 week	102 (14.41)	23 (28.75)	32 (20.51)	
2–4 week	101 (14.27)	12 (15.00)	21 (13.46)	
1–3 month	36 (5.08)	3 (3.75)	15 (9.62)	
> 3 month	26 (3.67)	7 (8.75)	11 (7.05)	
Bone marrow edema				0.005
Cranial type	119 (16.81)	5 (6.25)	34 (21.79)	
Diffused type	580 (81.92)	71 (88.75)	118 (75.64)	
Caudal type	9 (1.27)	4 (5.00)	4 (2.56)	
Lumbodarsal fascia edema				0.011
Detected	307 (43.36)	29 (36.25)	48 (30.77)	
Not detected	401 (56.64)	51 (63.75)	108 (69.23)	
Type of comorbidity				
Hypertension	345 (48.73)	37 (46.25)	76 (48.72)	0.914
Diabetes	128 (18.08)	9 (11.25)	23 (14.74)	0.220
Coronary heart disease	86 (12.15)	17 (21.25)	14 (8.97)	0.024
Cerebral infarction	146 (20.62)	29 (36.25)	29 (18.59)	0.003
Chronic pulmonary disease	25 (3.53)	3 (3.75)	6 (3.85)	0.979
Number of comorbidity				0.262
0	271 (38.28)	26 (32.50)	62 (39.74)	
1	230 (32.49)	28 (35.00)	48 (30.77)	
2	135 (19.07)	14 (17.50)	38 (24.36)	
3	58 (8.19)	10 (12.50)	8 (5.13)	
4	14 (1.98)	2 (2.50)	0 (0)	

*OVCF* osteoporotic vertebral compression fractures, *TL-OVCF* thoracolumbar OVCF, *nTL-OVCF* non-thoracolumbar OVCF, *MT* middle thoracic, *LL* lower lumbar

### nTL-OVCF in middle thoracic spine had higher comorbidity of coronary heart disease and cerebral infarction

Of the 944 OVCF 458 (48.5%) reported comorbidity of hypertension, 160 (16.9%) of diabetes, 117 (12.4%) of coronary heart disease, 204 (21.6%) of cerebral infarction, and 34 (3.6%) of chronic pulmonary disease. The middle thoracic OVCF had higher comorbidity of coronary heart disease (21.3%) and cerebral infarction (36.3%) than lower lumbar OVCF and TL-OVCF (Table 1). There were 359 (38.0%) reporting none, 306 (32.4%) with one, 187 (19.8%) with two, 76 (8.1%) with three, and 16 (1.7%) with four of the five common geriatric comorbidities. The number of comorbidity was not significantly different between nTL-OVCF and TL-OVCF (Table 1).

### nTL-OVCF experienced less apparent spine trauma and complained longer pre-hospital back pain

Of the 944 OVCF 580 (61.4%) reported apparent spine trauma, 172 (18.2%) with uncertain trauma, and 192 (20.3%) denied evident trauma to spine. The ratio of apparent spine trauma was 44.9% in nTL-OVCF and significantly lower than in TL-OVCF (66.9%) (Table 1). There were 555 (58.8%) OVCF hospitalized after complaining back pain for ≤ 1 week, 157 (16.6%) for 1–2 weeks, 134 (14.2%) for 2–4 weeks, 54 (5.7%) for 1–3 months, and 44 (4.6%) for > 3 months. The ratio of pre-hospital back pain for ≤ 1 week was 47.5% in nTL-OVCF and significantly lower than in TL-OVCF (62.6%) (Table 1).

### nTL-OVCF maintained vertebral compression degree with longer duration of pre-hospital back pain

**Fig. 3:**
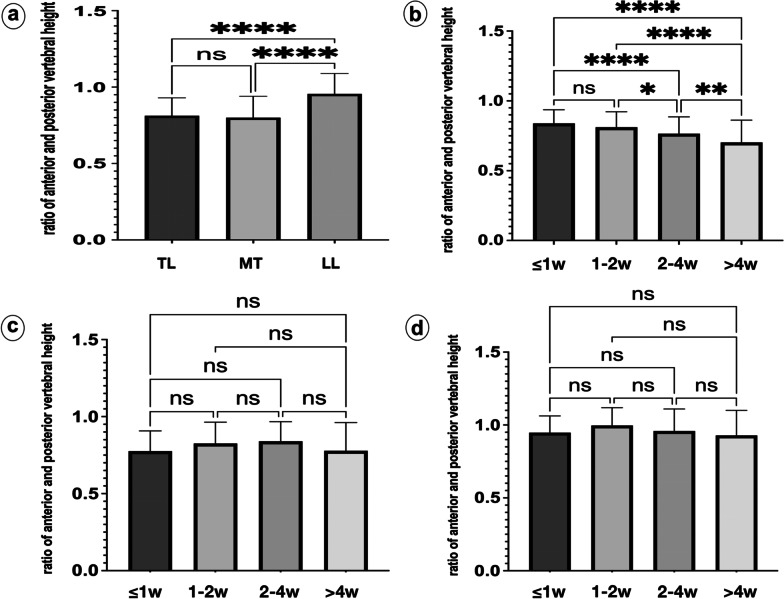
Comparison of vertebral compression degree. Comparison of the anterior–posterior vertebral height ratio in TL-OVCF and nTL-OVCF (**a-d**). The anterior–posterior vertebral height ratio in TL-OVCF and middle thoracic OVCF was significantly lower than in lower lumbar OVCF (**a**). The anterior–posterior vertebral height ratio of TL-OVCF was significantly lower with pre-hospital back pain for 2–4 and > 4 weeks than for ≤ 1 and 1–2 weeks (**b**). The anterior–posterior vertebral height ratio of nTL-OVCF in middle thoracic (**c**) and lower lumbar spine (**d**) differed not significantly with back pain for ≤ 1, 1–2, 2–4, and > 4 weeks. TL-OVCF: thoracolumbar OVCF; nTL-OVCF: non-thoracolumbar OVCF; MT: middle thoracic; LL: lower lumbar; w: weeks; ns: not significantly different; *P < 0.05; **P < 0.01; ****P < 0.001

The anterior–posterior vertebral height ratio was 0.81 ± 0.12 in TL-OVCF and significantly lower than in lower lumbar OVCF, but differed not significantly from that in middle thoracic OVCF (Fig. 3). In TL-OVCF, the anterior–posterior vertebral height ratio was 0.70 ± 0.16 with pre-hospital back pain for > 4 weeks and significantly lower than for 2–4 weeks, 1–2 weeks, and ≤ 1 week. In middle thoracic and lower lumbar OVCF, the anterior–posterior vertebral height ratio differed not significantly with pre-hospital back pain for ≤ 1, 1–2, 2–4, and > 4 weeks (Fig. 3).

### nTL-OVCF in lower lumbar spine had more cranial type of bone marrow edema and fewer concurrent lumbodorsal fasciitis

Of the 944 OVCF 769 (81.4%) showed diffused, 158 (16.7%) cranial, and 17 (1.8%) caudal type of vertebral bone marrow edema. The lower lumbar OVCF had higher ratio (21.8%) of cranial type of vertebral bone marrow edema than middle thoracic OVCF and TL-OVCF (Table 1). There were 384 (40.7%) OVCF showing edema signals in lumbodorsal fascia and diagnosed with concurrent lumbodorsal fasciitis. The lower lumbar OVCF had lower ratio (30.8%) of concurrent lumbodorsal fasciitis than middle thoracic OVCF and TL-OVCF (Table 1).

### nTL-OVCF and TL-OVCF had comparable bone mineral density

**Fig. 4:**
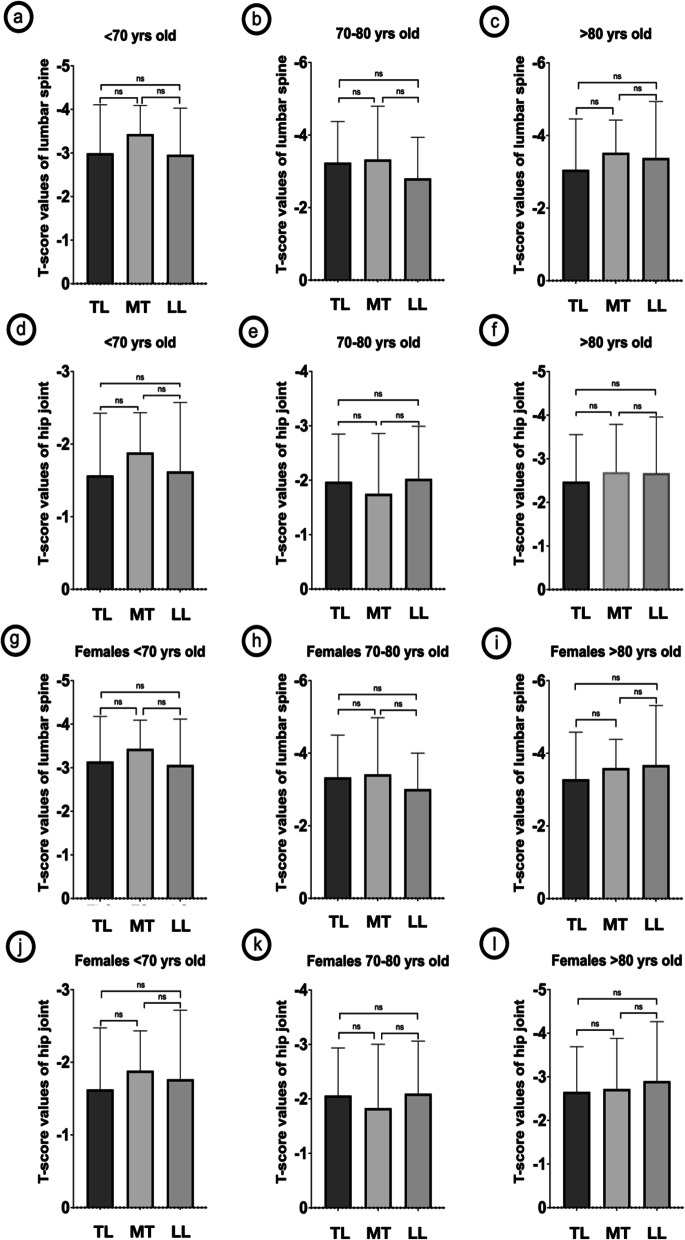
Comparison of bone mineral density. Comparison of the T-score values of lumbar spine (**a**-**c**, **g**-**i**) and hip joint (**d**-**f, j**-**l**) in TL-OVCF and nTL-OVCF. The T-score values showed no significant difference between TL-OVCF and nTL-OVCF aged < 70, 70–80, and > 80 years old (**a-f**). The T-score values of females showed no significant difference between TL-OVCF and nTL-OVCF aged < 70, 70–80, and > 80 years old (**g-l**). TL-OVCF: thoracolumbar OVCF; nTL-OVCF: non-thoracolumbar OVCF; MT: middle thoracic; LL: lower lumbar; yrs: years; ns: not significantly different

BMD was available from 371 (39.3%) OVCF and in 308 (40.1%) females. The T-score value of lumbar spine was − 2.99 ± 1.11, − 3.24 ± 1.14, − 3.05 ± 1.40 in < 70, 70–80, > 80 years old TL-OVCF, and differed not significantly from that in middle thoracic and lower lumbar OVCF (Fig. 4). The T-score value of hip joint was − 1.57 ± 0.86, − 1.97 ± 0.88, − 2.47 ± 1.08 in < 70, 70–80, > 80 years old TL-OVCF, and differed not significantly from that in middle thoracic and lower lumbar OVCF (Fig. 4). Of the 308 females, no significant difference was detected between TL-OVCF and nTL-OVCF in the BMD of lumbar spine or hip joint (Fig. 4).

## Discussion

As cement augmentation and spinal fixation potentially accelerate OVCF in adjacent vertebra [1, 11, 15], and multiple continuous OVCF often associate with high-energy trauma [16], the asymmetrical bimodal distribution of 944 single-segment OVCF without previous spine surgery may represent a nature distribution pattern of OVCF. We found that thoracolumbar vertebra had 2-folds higher risk of OVCF than non-thoracolumbar vertebra, supporting an increasing mechanical stress in the junction of thoracic and lumbar spine, peaking at L1 [5–9]. Moreover, we noticed that in the middle thoracic spine protected by rib cage, the incidence of OVCF was much lower than TL-OVCF, but still peaked at T7, suggesting increased mechanical stress in the apical vertebra of thoracic kyphosis [4]. In the lower lumbar vertebra with caudally increasing paravertebral muscular support [10, 17, 18], the incidence of OVCF decreased gradually from L1 to L5, suggesting absorption of the mechanical stress down along lumbar spine. In support of this notion, the lower lumbar OVCF had significantly higher ratio of cranial type of vertebral bone marrow edema than TL-OVCF and middle thoracic OVCF. The asymmetrical bimodal distribution pattern of single-segment OVCF could help to quantify the differences between thoracolumbar and non-thoracolumbar spine in the risk of mechanical injury and OVCF.

Despite increasingly recognized risk factors of vertebral fractures and re-fractures [11–15, 19, 20], it remains unknown the differences in the risk factors of TL-OVCF and nTL-OVCF. Here, we found that females had 3-folds higher risk of OVCF and experienced OVCF 3 years earlier than males. However, the female-male ratio was not significantly different between TL-OVCF and nTL-OVCF, suggesting a gender-independent anatomical distribution of OVCF. Aging causes bone fragility and potentiates OVCF [11–14]. We found that middle thoracic OVCF was comparatively older and had higher comorbidity of coronary heart disease and cerebral infarction than TL-OVCF, suggesting aging as risk factor for middle thoracic OVCF. Age-related bone mass loss and intervertebral disc degeneration increases thoracic kyphosis [21, 22], the increased middle thoracic OVCF in older patients might be attributed to an increased mechanical stress from thoracic hyperkyphosis [23, 24]. OVCF are most often caused by low-energy trauma [1, 2, 16]. Here, we found that 61.4% OVCF experienced apparent trauma such as fall on ground and crush injury to spine. However, just opposite to our hypothesis that nTL-OVCF might be associated with severer spine trauma, nTL-OVCF were shown to report lower ratio of apparent spine trauma than TL-OVCF. Comparison of BMD also identified no significant difference in the T-score values of lumbar spine or hip joint between TL-OVCF and nTL-OVCF. The independence of nTL-OVCF on female gender, apparent spine trauma, and poor BMD suggested the risk and distribution of TL-OVCF and nTL-OVCF might be determined mainly by the efficiency of mechanical loading, transmitting, and absorbing within specific segment and junction.

OVCF is often hospitalized with acute or chronic back pain [1–3]. Here, we found that the duration of pre-hospital back pain varied significantly from ≤ 1 week to > 3 months, and TL-OVCF tended to be hospitalized earlier than nTL-OVCF. Non-thoracolumbar spine is protected by rib cage and lumbar muscles, the delayed hospitalization of nTL-OVCF might be attributed to the paravertebral mechanical support that stabilizes vertebral compression to alleviate back pain [5, 9, 10]. In support of this notion, TL-OVCF rather than nTL-OVCF showed aggravation of vertebral compression with longer duration (> 2 weeks) of pre-hospital back pain. Evidence accumulates that lumbodorsal fascia is innervated and could be a potential source of back pain [25–27]. Here, we found a comorbidity rate of 40.7% of lumbodorsal fasciitis in acute single-segment OVCF without previous spine surgery. The higher rate of concurrent lumbodorsal fasciitis in TL-OVCF (43.3%) might aggravate back pain and promote early hospitalization.

Our study had several limitations. First, the design of retrospective study in single spine center would unavoidably yield bias in patient selection and data collection. The effects of pre-hospital bracing and analgesic medication [28, 29] on back pain and vertebral bone marrow edema were not evaluated. Secondly, BMD was unavailable from 60% of study population and in most of males, thus failing to fully understand the role of bone fragility in causing nTL-OVCF. Thirdly, radiographic parameters of regional or global kyphosis [20–23], body mass index [11, 14], anti-osteoporosis medication [14, 19, 30–32] and other risk factors of OVCF were not evaluated and compared between TL-OVCF and nTL-OVCF. Prospective studies with stratified participants and long-term follow-up are warranted for a better understanding of the segment-specific risk factors of OVCF.

## Conclusions

Single-segment OVCF demonstrates an asymmetrical bimodal distribution pattern showing 2-folds higher risk of TL-OVCF than nTL-OVCF. nTL-OVCF are not associated with female gender, apparent spine trauma or poor bone mineral density, but tend to maintain the degree of vertebral compression and cause longer duration of pre-hospital back pain.

## Data Availability

The datasets used and analysed during the current study are available from the corresponding author on reasonable request.

## Abbreviations

OVCF: Osteoporotic vertebral compression fractures
TL-OVCF: Thoracolumbar OVCF
nTL-OVCF: Non-thoracolumbar OVCF
BMD: Bone mineral density
